# Refractory gastroduodenal ulcers: A rare complication with Bloom syndrome

**DOI:** 10.1002/ccr3.6141

**Published:** 2022-09-12

**Authors:** Masaaki Usami, Yasuhiro Ikawa, Yuta Sakai, Toshihiro Fujiki, Taizo Wada

**Affiliations:** ^1^ Department of Pediatrics School of Medicine, Institute of Medical, Pharmaceutical and Health Sciences, Kanazawa University Kanazawa Japan

**Keywords:** Bloom syndrome, DNA repair disorder, gastroduodenal ulcer, gastroduodenostomy

## Abstract

Bloom syndrome patients often develop severe gastrointestinal symptoms mainly caused by gastric tumors due to DNA repair disorder. Here, we report 31‐year‐old Bloom syndrome patient suffering persistent abdominal pain due to refractory gastroduodenal ulcers which required gastroduodenectomy. Various causes should be considered, and the accumulation of their reports is warranted.

## Graphical abstract

We report a patient with Bloom syndrome who had refractory gastroduodenal ulcers requiring gastroduodenectomy. Since gastroduodenal ulcers with Bloom syndrome have not been reported so far, it was challenging to diagnose with her. As the complications of Bloom syndrome are diverse, the accumulation of their case reports is warranted.

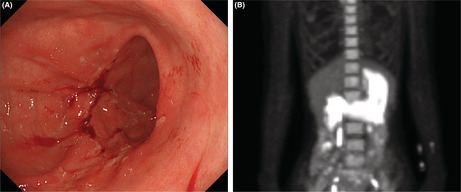

## 1 INTRODUCTION

Bloom syndrome is a rare congenital hereditary disorder of DNA repair due to an abnormality in RecQ helicase, resulting in various symptoms that include postnatal growth deficiency, sensitivity to sunlight, and immunodeficiency.^1^ It is also known to cause a high incidence of epithelial tumors in multiple organs, including the upper gastrointestinal tract. Therefore, careful management of gastrointestinal symptoms to rule out gastric cancers is required in affected people.^2^ Here, we report our experience of a patient with Bloom syndrome who had proton pump inhibitor (PPI)‐refractory epigastric pain. Various differential diagnoses were considered, including malignancy, infectious disease, and inflammatory bowel disease (IBD) due to the pathogenesis of Bloom syndrome^3^; however, there were no positive findings other than gastroduodenal ulcers. The patient finally required gastroduodenectomy. In recent years, PPIs have been widely administered for patients with gastroduodenal ulcers. The incidence of refractory gastroduodenal ulcers has decreased accordingly, and cases requiring gastroduodenectomy have become very rare.^4^ Clinicians who encounter cases of Bloom syndrome need to consider that these patients could suffer PPI‐refractory gastroduodenal ulcers that require gastroduodenectomy.

## 2 CASE REPORT

**FIGURE 1:**
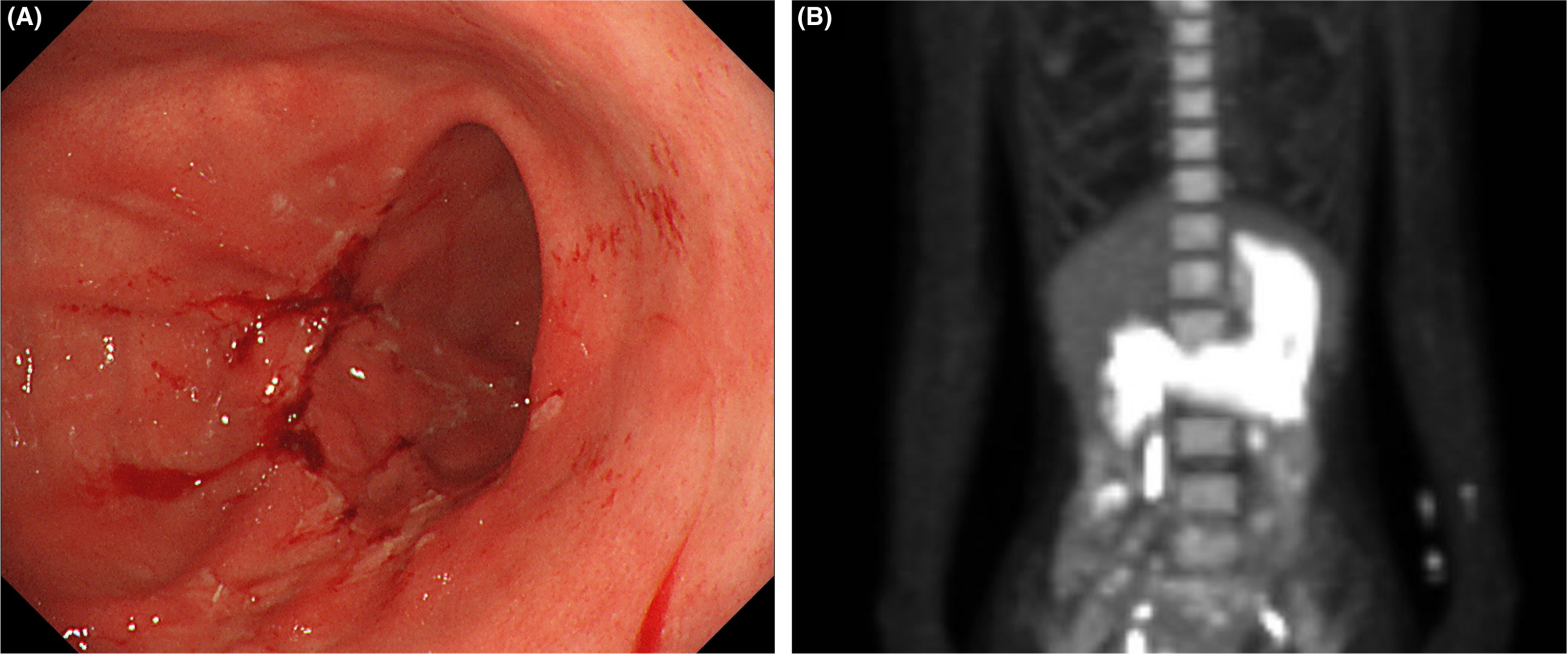
(A) Upper gastrointestinal endoscopic image. Gastric ulcers are seen on the anterior and posterior walls of the lower part of the gastric body. (B) FDG‐PET image reveals intense uptake by the entire gastroduodenal wall. Abbreviations: FDG‐PET, ^18^F‐fluorodeoxyglucose positron emission tomography

**FIGURE 2:**
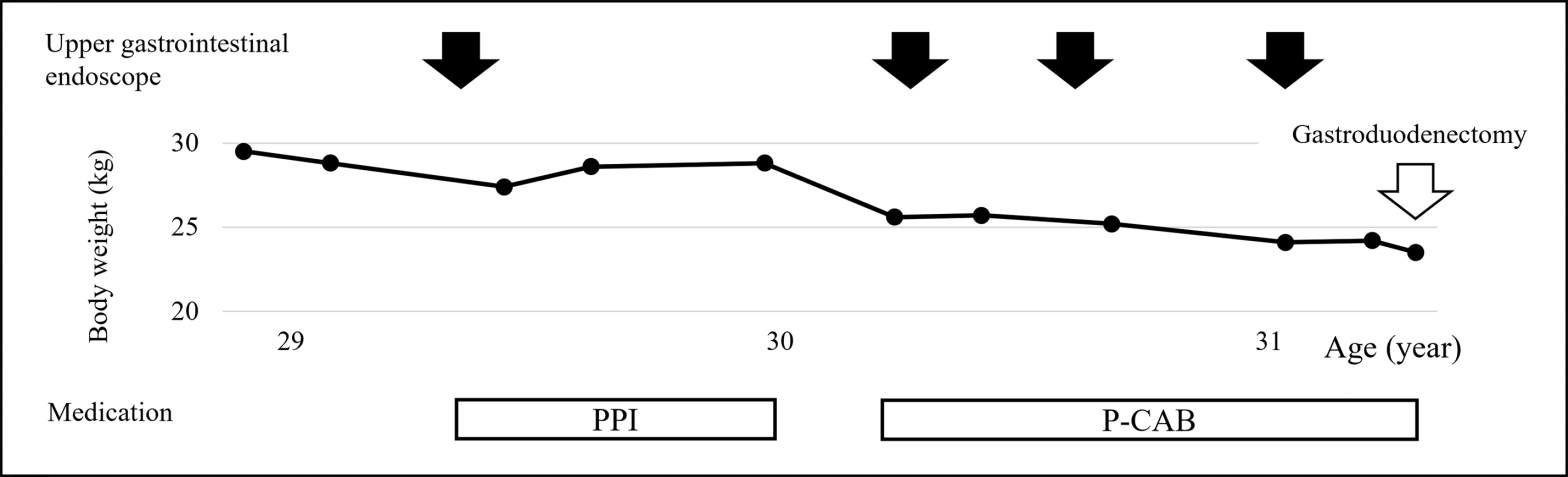
Longitudinal follow‐up of the patient's body weight. Abbreviations: PPI, proton pump inhibitor; P‐CAB, potassium‐competitive acid blocker

**FIGURE 3:**
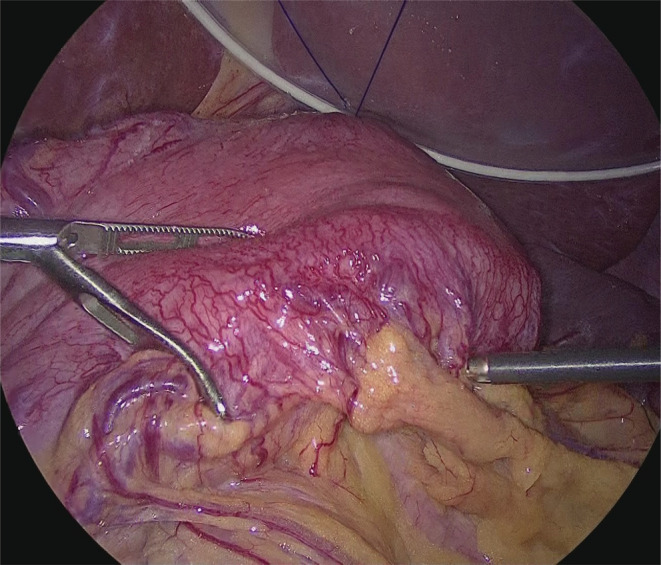
Image obtained during laparoscopic surgery. Neovascularization and thickening of the intestinal wall are observed on the ventral side of the gastroduodenum

**FIGURE 4:**
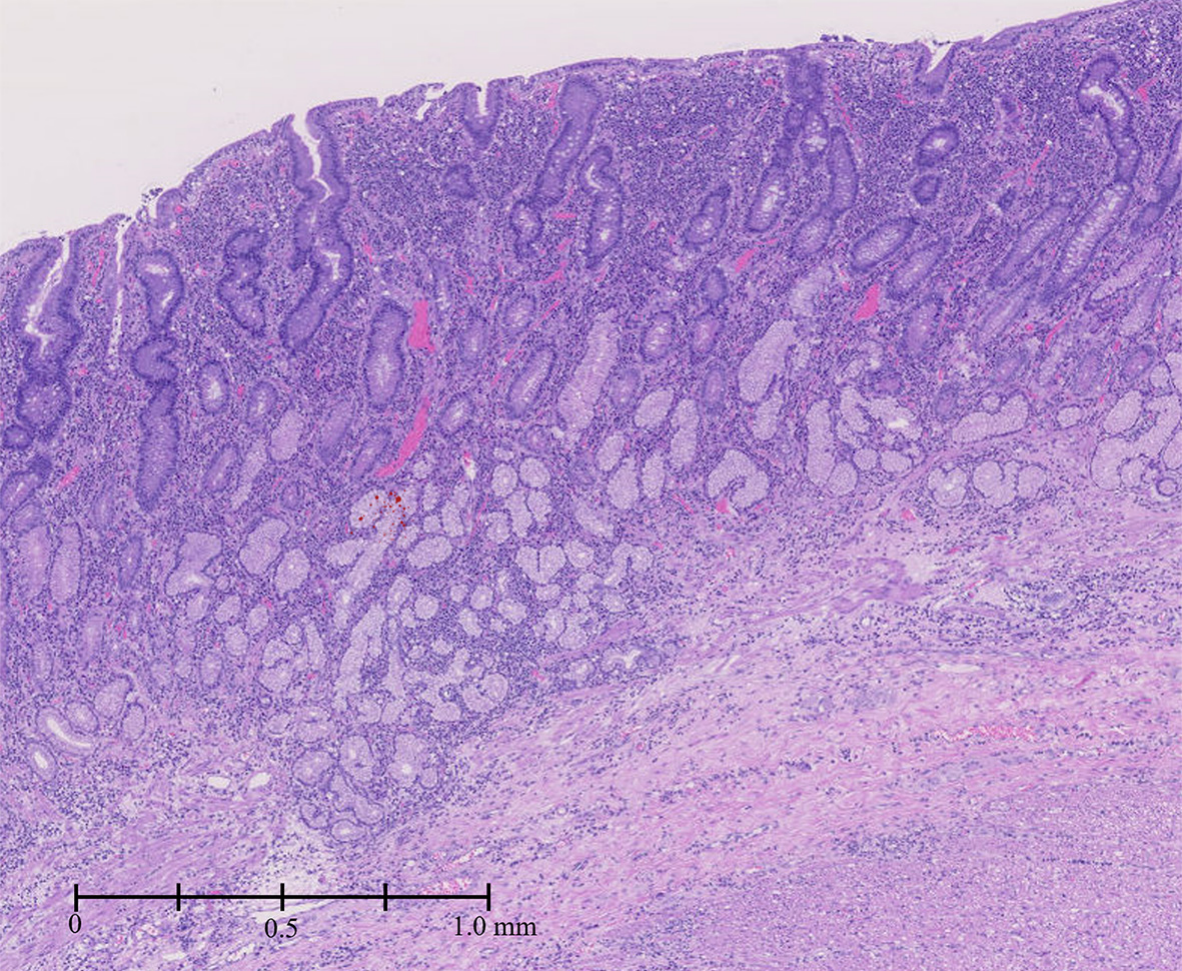
Hematoxylin and eosin stain image of the resected stomach tissue. Numerous inflammatory cell infiltrates were observed in the gastric mucosa

A 31‐year‐old woman was admitted to our hospital due to persistent epigastric pain. She had been diagnosed with Bloom syndrome at the age of 6 years based on clinical features such as short stature, café‐au‐lait spots, and hypogammaglobulinemia. At the age of 23 years, she developed intermittent abdominal pain, anorexia, and weight loss, and was diagnosed with primary peripheral T‐cell lymphoma, not otherwise specified (PTCL‐NOS) in the ileum (Stage 1). She underwent resection of the malignant lesion and intensive chemotherapy (CHOP regimen). No recurrence of the tumor has been observed so far. At the age of 29 years, she began to complain of epigastric pain. An upper gastrointestinal endoscopy was performed, and duodenal ulcers (stage A1 of the Sakita–Miwa classification) and superficial gastritis were found, with no findings of malignancy. The symptoms were relieved by an oral PPI, but the abdominal pain reappeared a few months later. Repeat endoscopic examinations revealed multiple ulcers in the stomach and duodenum (Figure 1A). Based on the possibility of recurrence of malignancy or complications of IBD, lower gastrointestinal endoscopy was also performed, but there were no obvious abnormal mucosal findings. Various medications were administered accordingly, including potassium‐competitive acid blocker (P‐CAB), but there was no improvement in symptoms. Because of her high risk of carcinogenesis due to Bloom syndrome, it was also necessary to rule out gastrinoma. Her gastrin level was high enough to suspect gastrinoma (1341 pg/mL; standard range, 42–200 pg/mL). ^18^F‐fluorodeoxyglucose positron emission tomography (FDG‐PET) showed intense accumulation of FDG in the entire gastroduodenum although somatostatin receptor scintigraphy showed no accumulation (Figure 1B). Repeated gastroduodenal histopathology showed negative results for malignancy including gastrinoma. The calcium tolerance test did not show an increase in serum gastrin level of >20%, suggesting that the patient was probably negative for gastrinoma. In the differential diagnosis among other immune deficiency diseases, Epstein–Barr virus, cytomegalovirus, herpes simplex virus, and *Helicobacter pylori* were considered, but all were ruled out by antibody titer of serum and fluorescent staining of histopathology. As the patient's food intake and body weight had gradually decreased despite taking various medications (Figure 2), we decided to perform total gastroduodenal resection. Intraoperatively, massive neovascularization and thickening of the intestinal wall were observed on the ventral gastroduodenum (Figure 3), indicating chronic inflammation. The pathological findings revealed increased inflammatory cells in the gastroduodenal mucosa, but no neoplastic lesions or granulomas suggestive of Crohn's disease (Figure 4). Based on these results, refractory gastric ulcers might be considered a complication associated with Bloom syndrome. After the surgery, her food intake recovered, and the abdominal pain improved.

## 3 DISCUSSION

We experienced a case of Bloom syndrome with refractory gastroduodenal ulcers that required total gastroduodenectomy. As Bloom syndrome is characterized by impaired gene repair, the patient's gastrointestinal symptoms led us to rule out the development of epithelial malignancies. It was also necessary to rule out various other infections due to immunodeficiency, including Epstein–Barr virus, cytomegalovirus, and herpes simplex virus.^5^ Recent studies have reported the development of IBD in patients with DNA repair defects,^6^ including ulcerative colitis in patients with Bloom syndrome.^3^ Although the exact mechanism of the development of IBD is not understood, chromosomal stability is considered to be a factor that may affect the development of IBD.^7^ Therefore, when a patient with Bloom syndrome presents with refractory gastroduodenal ulcers, the possibility of a neoplastic lesion, infectious disease related to immunodeficiency, or IBD should be considered.

It is important to note that few phenotypes of this rare syndrome have been described so far. According to the *Bloom Syndrome Registry,* there are fewer than 300 patients worldwide with Bloom syndrome.^8^ Because reports of gastrointestinal diseases associated with Bloom syndrome are particularly limited, it is often challenging to obtain a differential diagnosis based on their symptoms and the clinical examination.^2^ Therefore, the accumulation of cases is essential for further investigation of its pathogenesis and treatment.

In conclusion, we experienced a patient with Bloom syndrome who had PPI‐refractory gastroduodenal ulcers requiring gastroduodenectomy. As the complications of Bloom syndrome are diverse, it is essential to perform appropriate diagnostic examinations and careful follow‐up.

## AUTHOR CONTRIBUTIONS

M.U. wrote the manuscript, and Y.S. and T.F. provided patient care. Y.I. was the principal investigator and takes primary responsibility for the paper. T.W. reviewed and provided advice on this manuscript. All authors have read and approved the final manuscript.

## CONFLICT OF INTEREST

The authors declare that there are no conflicts of interest.

## CONSENT

Written informed consent was obtained from the patient to publish this report in accordance with the journal's patient consent policy.

## Data Availability

The data that support the findings of this study are available on request from the corresponding author. The data are not publicly available due to privacy or ethical restrictions.
